# Exosomes/tricalcium phosphate combination scaffolds can enhance bone regeneration by activating the PI3K/Akt signaling pathway

**DOI:** 10.1186/s13287-016-0391-3

**Published:** 2016-09-20

**Authors:** Jieyuan Zhang, Xiaolin Liu, Haiyan Li, Chunyuan Chen, Bin Hu, Xin Niu, Qing Li, Bizeng Zhao, Zongping Xie, Yang Wang

**Affiliations:** 1 Institute of Microsurgery on Extremities, Shanghai Jiao Tong University Affiliated Sixth People’s Hospital, 600 Yishan Road, Shanghai, 200233 China; 2 Department of Orthopedic Surgery, Shanghai Jiao Tong University Affiliated Sixth People’s Hospital, 600 Yishan Road, Shanghai, 200233 China; 3 Med-X Research Institute, School of Biomedical Engineering, Shanghai Jiao Tong University, 1954 Huashan Road, Shanghai, 200030 China; 4 Graduate School of Nanchang University, 461 Bayi Road, Nanchang, 330006 China

**Keywords:** iPS-MSCs, Exosomes, Tricalcium phosphate, Bone regeneration, Microarray, PI3K/Akt

## Abstract

**Background:**

Recently, accumulating evidence has shown that exosomes, the naturally secreted nanocarriers of cells, can exert therapeutic effects in various disease models in the absence of parent cells. However, application of exosomes in bone defect repair and regeneration has been rarely reported, and little is known regarding their underlying mechanisms.

**Methods:**

Exosomes derived from human-induced pluripotent stem cell-derived mesenchymal stem cells (hiPS-MSC-Exos) were combined with tricalcium phosphate (β-TCP) to repair critical-sized calvarial bone defects, and the efficacy was assessed by histological examination. We evaluated the in vitro effects of hiPSC-MSC-Exos on the proliferation, migration, and osteogenic differentiation of human bone marrow-derived mesenchymal stem cells (hBMSCs) by cell-counting, scratch assays, and qRT-PCR, respectively. Gene expression profiling and bioinformatics analyses were also used to identify the underlying mechanisms in the repair.

**Results:**

We found that the exosome/β-TCP combination scaffolds could enhance osteogenesis as compared to pure β-TCP scaffolds. In vitro assays showed that the exosomes could release from β-TCP and could be internalized by hBMSCs. In addition, the internalization of exosomes into hBMSCs could profoundly enhance the proliferation, migration, and osteogenic differentiation of hBMSCs. Furthermore, gene expression profiling and bioinformatics analyses demonstrated that exosome/β-TCP combination scaffolds significantly altered the expression of a network of genes involved in the PI3K/Akt signaling pathway. Functional studies further confirmed that the PI3K/Akt signaling pathway was the critical mediator during the exosome-induced osteogenic responses of hBMSCs.

**Conclusions:**

We propose that the exosomes can enhance the osteoinductivity of β-TCP through activating the PI3K/Akt signaling pathway of hBMSCs, which means that the exosome/β-TCP combination scaffolds possess better osteogenesis activity than pure β-TCP scaffolds. These results indicate that naturally secreted nanocarriers-exosomes can be used as a bioactive material to improve the bioactivity of the biomaterials, and that hiPS-MSC-Exos combined with β-TCP scaffolds can be potentially used for repairing bone defects.

**Electronic supplementary material:**

The online version of this article (doi:10.1186/s13287-016-0391-3) contains supplementary material, which is available to authorized users.

## Background

The effective reconstruction of bone defects is a challenging problem in orthopedic surgery. Autologous and allogenic bone grafting are the most widely used treatments for bone defect repair. However, the limited availability of graft material, the extra damage to harvest sites, and the frequent need for a second operation suppressed autologous bone grafts [1, 2]; allogenic bone grafts are hampered by problems of significant failure rates, poor mechanical stability, and immunological rejection [3]. Bioactive materials provide an alternative solution for the repair and regeneration of bone defects. Tricalcium phosphate (β-TCP), composed of the same ions as bone, is a well characterized osteoconductive biomaterial and is already in clinical use for bone repair and regeneration [4, 5]. However, β-TCP is largely considered to lack osteoinductive activity, which may impact its repair capacity for large bone defects and nonunions [6–8].

Mesenchymal stromal cells (MSCs) have been widely studied for tissue repair and regeneration [9, 10]. Recently, accumulating evidence has revealed that the positive effects of MSCs on tissue repair are not facilitated by the direct differentiation into the parenchymal cell that repairs and replaces the damaged tissues, but rather by stimulating the activity of tissue-resident recipient cells via paracrine mechanisms [11–13]. Exosomes, the naturally secreted nanocarriers from cells, are 40–150 nm nanoparticles originating from multivesicular bodies, and play key roles in intercellular communication by transferring proteins and genetic information to target cells [14]. It has been reported that MSC-derived exosomes have therapeutic potential in various disease models in the absence of MSCs [15–17]. In addition, exosomes have been reported to be able to exhibit similar functional properties to the cells from which they are derived and have no apparent adverse effects [18–20]. Therefore, exosomes derived from MSCs may also play a critical role in tissue regeneration.

However, collecting enough exosomes for tissue repair relies on abundant parent cell sources. Recent studies indicate that MSC-like cells derived from iPSCs (iPS-MSCs) offer the advantages of both MSCs and iPSCs. Specifically, abundant MSCs can be generated from iPSCs that can be passaged >40 times in culture and still sustain the self-renewal capacity characteristic of MSCs [21]. iPS-MSCs also possess greater proliferative capacity and immunoregulatory function compared with adult MSCs [21, 22]. In addition, a previous study demonstrated that iPS-MSCs had bone regeneration ability when they were directly transplanted into calvarial defects in immunocompromised mice [23]. These features make iPS-MSCs an excellent cell source for exosome generation. Considering the similar therapeutic effects of exosomes to those of their parent cells, we hypothesize that the exosomes derived from iPS-MSCs may also have pro-osteogenesis ability. Thus, we propose to use exosomes to functionalize biomaterials and obtain an exosome/biomaterial combination in order to improve the osteogenesis ability of biomaterials.

Therefore, we used human iPS-MSCs as cell sources to generate exosomes (hiPS-MSC-Exos) and investigated whether these naturally secreted nanocarriers could enhance bone repair and the regeneration ability of β-TCP. We combined hiPS-MSC-Exos with β-TCP scaffolds and we verified that hiPS-MSC-Exos/β-TCP combination scaffolds could significantly promote osteogenesis in a rat model of critical-sized calvarial bone defect as compared to β-TCP alone. In vitro studies, including the release of exosomes from hiPS-MSC-Exos/β-TCP combination scaffolds and internalization of exosomes into hBMSCs, have been conducted. After internalization, the proliferation, migration, and osteogenic differentiation of hBMSCs have been investigated. By using gene expression profiling and bioinformatics analyses, we have proved that the exosomes can activate the PI3K/Akt signaling pathway, which may be one of the underlying mechanisms through which hiPS-MSC-Exos/β-TCP combination scaffolds stimulate osteogenesis as compared to β-TCP alone.

## Methods

### Generation and characterization of hiPS-MSCs

A comprehensive method description and results on the derivation and characterization of hiPS-MSCs have been provided in our previous publications [13, 24]. Briefly, the human iPS cells were routinely cultured and expanded on ESC-Qualified BD Matrigel (BD Biosciences, Sparks, USA) in six-well plates in mTESR1 (StemCell Technologies, Vancouver, Canada). When cells were 90 % confluent, mTESR1 was replaced by low-glucose Dulbecco’s modified Eagle medium (Corning, Tewksbury, USA) containing 10 % fetal bovine serum (FBS; Gibco, Grand Island, USA) and 2 mM l-Glutamine (Gibco, Grand Island, USA). The medium was changed every other day. After 14 days in culture, the cells were trypsinized and resuspended in MSC media and plated onto 0.1 % gelatin-precoated dishes. Cells were passaged when they reached 80–90 % confluency.

Usually, cells at passage 4 exhibited a typical MSC-like morphology and were used for cell surface marker identification by flow cytometry analysis. Briefly, 5 × 10^5^ cells were fixed with 4 % paraformaldehyde for 15 min, blocked with 3 % BSA in PBS for 30 min, and incubated with the following primary antibodies (BD Biosciences): CD29, CD34, CD73, CD90, CD105, and HLA-DR. Nonspecific fluorescence was determined by incubation of similar cell aliquots with isotype-matched mouse monoclonal antibodies. The cells were washed with PBS and analyzed using the Guava easyCyte™ Flow Cytometer (Millipore, Billerica, MA, USA).

Tri-lineage differentiation potential was confirmed by qRT-PCR measurement of gene expression levels of markers associated with osteo- (OCN), chondro- (Sox9), and adipogenic (LPL) differentiation after 7 days in culture with osteo-, chondro-, and adipogenic mediun.

### Isolation and identification of hiPS-MSC-Exos

When hiPS-MSCs were 80–90 % confluent, the culture medium was replaced by the MGro-500 chemically defined serum-free MSC medium (StemRD), and the cells were cultured for an additional 48 h. The conditioned medium (CM) of hiPS-MSCs was obtained and centrifuged at 300 × g for 10 min and at 2000 × g for 10 min to remove dead cells and cellular debris. Then, the supernatant was filtered through a 0.22-μm filter sterilizer (Steritop TM; Millipore, Billerica, USA) to remove the residual cellular debris. Subsequently, the supernatant was centrifuged at 4000 × g to about 200 μL by ultra-filtration in a 15 mL Amicon Ultra-15 Centrifugal Filter Unit (Millipore, Billerica, USA). The ultra-filtrated liquid was washed twice with 15 mL PBS and re-ultra-filtrated at 4000 × g to 200 μL. For exosome purification, the liquid was overlaid onto a 30 % sucrose-D_2_O cushion in a sterile Ultra-Clear™ tube (Beckman Coulter, Kraemer Boulevard Brea, USA) and ultra-centrifuged at 100,000 × g for 2 h to pellet the small vesicles that correspond to exosomes. The pelleted exosomes were resuspended in 15 mL PBS and centrifuged at 4000 × g in Centrifugal Filter Units until the final volume was reduced to approximately 200 μL. All procedures were performed at 4 °C. Exosomes were stored at –80 °C or used for downstream experiments. Transmission electron microscopy (TEM), tunable resistive pulse sensing (TRPS) analysis, and western blotting were used to identify hiPS-MSC-Exos.

### In vivo animal experiments

#### Surgical procedures

All procedures were approved by the Animal Research Committee of the Sixth People’s Hospital, Shanghai Jiao Tong University, China. Classical porous tricalcium phosphate (β-TCP) scaffolds with a dimension of 5 mm in diameter and 2 mm in depth (Bio-lu Biomaterials Co. Ltd., Shanghai) with an average pore size of 500 μm and 75 % porosity were used as exosome carriers in the in vivo studies. Five × 10^11^ particles/mL or 1 × 10^12^ particles/mL of exosomes (100 μL) or an equal volume of exosome diluent (PBS) were blotted onto each β-TCP scaffold under sterile conditions and left still for at least 4 h for the exosomes to be totally absorbed. Sprague-Dawley (SD) rats weighing 250–300 g were anesthetized, and two critical-sized calvarial defects with a diameter of 5 mm were created on each side of the cranium using dental trephine followed by implantation of the scaffolds into the defects. Eighteen rats were randomly divided into three groups for the following implants: (1) β-TCP (*n* = 6); (2) β-TCP + Exos (5 × 10^11^ particles/mL) (*n* = 6); and (3) β-TCP + Exos (1 × 10^12^ particles/mL) (*n* = 6). At 8 weeks after surgery, the rats were sacrificed and the craniums were harvested. Craniums were first analyzed by micro-CT. Then, every cranium was divided into two halves, one for non-decalcified histological analysis and the other for decalcified immunohistochemical (IHC) analysis.

#### Sequential fluorescent labeling

At 2, 4, and 6 weeks after the operation, all the rats were intraperitoneally injected with tetracycline (25 mg/kg body weight), alizarin red (30 mg/kg body weight), and calcein (20 mg/kg body weight), respectively. The trichromatic sequential fluorescent assessment was performed to observe the mineralized tissue.

#### Micro-CT analysis

At 8 weeks after the operation, every rat was euthanized, and craniums were then explanted and fixed in 10 % neutral buffered formalin solution for longer than 48 h. The craniums were scanned using micro-CT (Skyscan 1176, Kontich, Belgium) at 18-μm resolution. Three-dimensional grayscale images were generated using the CTVol program. As there are density differences between β-TCP and new bone, CTAn software used in this study can distinguish one from the other. Then, percentage of new bone volume relative to tissue volume (BV/TV) and bone mineral density (BMD) in the bone defect were both calculated.

#### Histological and IHC analysis

Half of every fixed cranium was dehydrated through graded alcohol series, and embedded in polymethylmethacrylate (PMMA). After hardening, the sagittal sections of the central segment were cut into 150–200-μm thick slices using a microtome (Leica, Hamburg, Germany) and glued onto a plastic support and polished to a final thickness of approximately 40 μm. Subsequently, a confocal laser scanning microscope (Leica, Heidelberg, Germany) was used to observe fluorescent labeling of the sections with chelating fluorochromes employing the excitation/emission wavelengths of 405/580 nm for tetracycline (yellow), 543/617 nm for alizarin red (red), and 488/517 nm for calcein (green). The sections were stained with van Gieson’s picrofuchsin and were observed with a microscope (Leica, Solms, Germany) to assess new bone formation. In the image, red and black indicated new bone and β-TCP, respectively. The other half of the craniums was decalcified for 2 to 3 weeks in 10 % EDTA solution, dehydrated with gradient alcohols, embedded in paraffin, and then sectioned into 4-mm thick sections. Osteogenesis was evaluated by IHC analysis for osteocalcin (OCN).

### In vitro studies

#### Cell culture

Human BMSCs (hBMSCs) were obtained from four donors (the donors at an average age of 35 are healthy in daily life, but are amputees due to trauma) with written informed consent. Briefly, the marrow of the femora midshaft was extracted and then suspended in α-MEM containing 10 % fetal bovine serum (FBS; Hyclone), 100 U/mL penicillin, and 100 mg/mL streptomycin (Hyclone). Non-adherent cells were removed and adherent cells were passaged after becoming 80 % confluent. Early-passage BMSCs (p2–6) were used in the experiments as described below.

### Exosome release from β-TCP

hiPS-MSCs were labeled with Vybrant DiO (green) dye (Molecular Probes, Carlsbad, CA, USA) according to the manufacturer’s protocol. Briefly, the cells were trypsinized and resuspended in 1 mL MGro-500 serum-free MSC media; 5 μL of the cell-labeling solution was added to the cells, followed by incubation at 37 °C and 5 % CO_2_ for 15 min. The labeled cell suspension was centrifuged at 300 × g for 15 min and the supernatant was discarded. The cells were washed three times with PBS and then cultured in MSC media for an additional 24 h. To detect exosome release from β-TCP, exosomes isolated and purified from the hiPS-MSC-derived CM were combined with β-TCP and then added to the 24-well plate when hBMSCs were about 80–90 % confluent. After culturing for 8 h in this medium, the exosome/β-TCP combination was removed and added to another well. The hBMSCs were washed twice with PBS, fixed with 4 % paraformaldehyde for 15 min, and stained with DAPI for 5 min at room temperature. After that, the cells were analyzed with a fluorescence microscope (Leica DMI6000B, Solms, Germany) to observe the release of exosomes. The detection of exosomes released from β-TCP was carried out for 2 days.

The release rate of exosomes from TCP was also detected by TRPS analysis. Briefly, 1 × 10^12^ particles of exosomes were combined with β-TCP and then added to the 24-well plate supplemented with 200 μl PBS. After culturing for 1 day, the PBS was collected and refilled with 200 μl fresh PBS. The detection was carried out for 5 days. The particles in PBS were finally counted by TRPS analysis.

### Cell proliferation assay

A Cell Counting Kit-8 assay (CCK-8; Dojindo, Kyushu Island, Japan) was performed to assess cell proliferation as previously described [7]. Briefly, hBMSCs (5 × 10^3^ cells per well) were seeded onto 96-well plates and cultured at 37 °C in MSC media supplemented with 5 × 10^11^ or 1 × 10^12^ particles/mL of exosomes. Cells treated with an equal volume of PBS served as controls, and a group without cells served as the blank. At day 1, 2, 3, 4, and 5, CCK-8 solution (10 μL per well) was added to hBMSCs and subsequently incubated at 37 °C for 3 h. The absorbance was measured at 450 nm using a microplate reader. The optical density (OD) values represented the survival/proliferation of hBMSCs.

### Migration assay

Cell migration was assessed using a scratch wound healing assay as previously described [24]. Briefly, hBMSCs (1 × 10^5^ cells per well) were plated in 12-well plates and incubated at 37 °C. After cells had attached, the confluent monolayer was scratched using a p200 pipette tip and washed with PBS to remove the debris and smooth the edge of the scratch; 1 mL of MSC medium supplemented with exosomes (5 × 10^11^ or 1 × 10^12^ particles/mL) or an equal volume of PBS was added to each well. The cells were photographed immediately (t = 0 h), and 12 h (t = 12 h) and 24 h (t = 24 h) later using a microscope (Leica DMI6000B, Solms, Germany). The migration area (%) was assessed as follows: migration area (%) = (A_0_ – A_n_)/A_0_ × 100, where A_0_ represents the initial wound area (t = 0 h) and A_n_ represents the residual area of the wound at the metering point (t = n h).

### Gene expression profiling and bioinformatics analysis

hBMSCs were seeded in a 25-cm^2^ cell culture flask and cultured in MGro-500 serum-free MSC medium containing exosomes (5 × 10^11^ particles/mL) or an equal volume of PBS at 37 °C, 5 % CO_2_ for 24 h. Gene expression profiling analyses of hBMSCs were performed by using the GeneChip® PrimeView™ Human Gene Expression Array (Affymetrix, Santa Clara, CA, USA), which contained approximately 20,000 annotated *Homo sapiens* gene probes. The experiments were performed in triplicate. A *p* value cut-off of 0.05 and a fold-value change of ≥2 were used as a filter to identify the differentially expressed (DE) genes. The expression values of differentially expressed genes were transformed to Z-scores and then hierarchically clustered based on Euclidean distance and average-linkage. Then, a gene set enrichment analysis of all differentially expressed genes was performed based on the Database for Annotation as well as Visualization and Integrated Discovery (DAVID) to identify the significantly enriched pathways. The 1.5-fold up- or downregulated genes in the selected enriched pathways were then illustrated as a heat map.

### Quantitative real-time PCR analysis (qRT-PCR)

The expression levels of selected differentially expressed genes after the functional enrichment analysis were confirmed by qRT-PCR. Total RNAs were extracted using Trizol Reagent (Invitrogen, USA) and reverse-transcribed into complementary (c)DNAs using the RevertAid first-strand cDNA synthesis kit (Fermentas, Life Sciences, Burlington, Canada). The qRT-PCR analysis was performed using an ABI PRISM®7900HT System (Takara Biotechnology, Japan). GAPDH was employed as the housekeeping gene for internal normalization. Primers used in the amplification reaction are listed in Additional file 1 (Table S1).

### Western blot analysis

Western blotting was performed as described previously [7]. Cells or exosome lysates were diluted at a ratio of 1:4 with protein loading buffer (5×) and heated at 95 °C for 5 min. Protein extracts were separated on a 10 % sodium dodecyl sulfate-polyacrylamide gel electrophoresis (SDS-PAGE) gel at 120 V for 1 h and blotted onto nitrocellulose membranes (Whatman, Maidstone, Kent, UK) for 30 min at 100 mA. The membranes were then blocked for 2 h with 5 % non-fat dried milk in TBST (10 mM Tris-HCl pH 7.5, 150 mM NaCl, 0.1 % Tween-20). Subsequently, the membranes were incubated with primary antibodies at 4 °C overnight, followed by incubation with horseradish peroxidase (HRP)-labeled secondary antibodies (Cell Signaling Technology, USA) at 37 °C for 1 h. The primary antibodies including anti-CD9, anti-CD63, anti-CD81, anti-OCN, anti-runt-related transcription factor 2 (Runx2), anti-collagen type I alpha 1 (COL1A1), anti-Akt, and phosphorylated Akt (p-Akt) antibody were obtained from Abcam (Cambridge, UK). The immunoreactive bands were visualized using enhanced chemiluminescence reagent (Thermo Fisher Scientific, Waltham, MA, USA) and imaged by an Image Quant LAS 4000 mini bio-molecular imager (GE Healthcare, Uppsala, Sweden). All values were normalized to the value of beta-actin (β-actin).

### Alkaline phosphatase (ALP) assay and Alizarin red S (ARS) staining

hBMSCs were cultured in osteogenic media supplemented with exosomes or PBS for 10 days (ALP staining) or 14 days (ARS staining). Cells were washed with PBS, fixed with 4 % paraformaldehyde for 10 mins, and incubated with the ALP reagents (Sigma) or with 2 % ARS solution (Sigma) for 30 min at 20 °C according to the manufacturer’s protocol. After washing with distilled water, the stained cells were examined using an inverted microscope (Leica DMI6000B, Solms, Germany). ALP activity of hBMSCs was measured using a commercial kit (Sigma) following the manufacturer’s instructions.

### PI3K/Akt signaling inhibition

LY294002, a highly selective inhibitor of PI3K, was purchased from Sigma-Aldrich (St. Louis, MO, USA) and dissolved in dimethylsulfoxide (DMSO) at a stock concentration of 100 mM according to the manufacturer’s protocol. To confirm the involvement of PI3K/Akt signaling in the exosome-mediated effects on hBMSCs, cells were pre-treated with the indicated concentration (10 μM) of LY294002 or an equal volume of DMSO for 1 h. Subsequently, 5 × 10^11^ particles/mL of exosomes or an equal volume of PBS was added to the culture medium of hBMSCs and the cells were cultured for 24 h. Western blotting, ALP, and ARS staining on hBMSCs were then performed as described above.

### Statistical analysis

All data are shown as mean ± standard deviation (SD). Differences between groups were assessed by one-way analysis of variance (ANOVA); Dunnett t or LSD t analyses were performed to determine the statistical significance between each sample. *P* < 0.05 was considered statistically significant.

## Results

### Characterization of hiPS-MSCs and hiPS-MSC-Exos

**Fig. 1:**
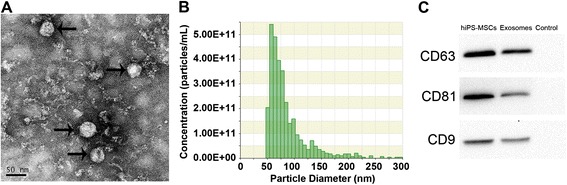
Characterization of exosomes derived from human induced pluripotent stem cell-derived mesenchymal stem cells (*hiPS-MSCs*). **a** Morphology observed by TEM. **b** Particle size distribution and concentration measured by TRPS. **c** Western blot analysis of the exosome surface markers

Using a modified one-step induction protocol [25], almost 100 % of the hiPS cells successfully differentiated into hiPS-MSCs, and the hiPS-MSCs exhibited characteristics of MSCs (Additional file 1: Figure S1). TEM, TRPS analysis, and western blotting were used to characterize the purified nanocarriers derived from hiPS-MSCs. The results showed that the vast majority of these particles exhibited a cup- or round-shaped morphology with a size ranging from 50 to 150 nm (Fig. 1a and b), suggesting the presence of exosomes. Also, these particles did express the characteristic exosome surface markers, including CD9, CD63, and CD81 (Fig. 1c), which further confirmed their exosome identity. The data suggested that these nanocarriers were predominantly exosomes.

### In vivo osteogenesis activity of exosome/β-TCP combination scaffolds

**Fig. 2:**
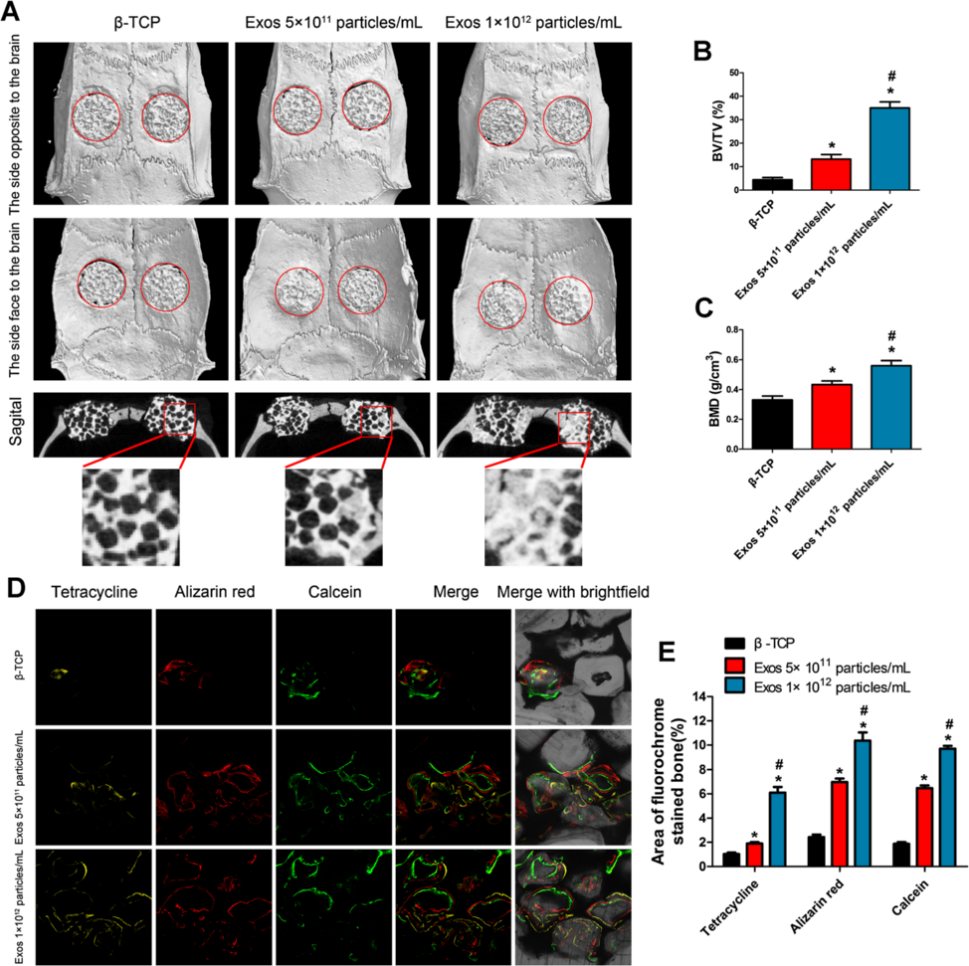
Micro-CT and fluorochrome-labeling histomorphometrical analysis of the repaired craniums at 8 weeks post-implantation. **a** Three-dimensional reconstruction and sagittal images show the different reparation effects of exosomes or tricalcium phosphate (*β-TCP*) only. **b** The bone volume/tissue volume (*BV/TV*) and **c** bone mineral density (*BMD*) varied between the different groups. **d** Fluorochrome-labeling histomorphometrical analysis of new bone formation and mineralization at 8 weeks post-operation (*yellow*, between 2 and 4 weeks; *red*, between 4 and 6 weeks; *green*, between 6 and 8 weeks). **e** Percentage of each fluorochrome area for different groups. Dunnett t test; *p < 0.05 compared with the β-TCP group, ^#^ *p* < 0.05 compared with the exosome (*Exos*) 5 × 10^11^ particles/mL group

To investigate the therapeutic potential of hiPS-MSC-Exos on bone repair, the rat critical-sized calvarial bone defect model was established and the exosome/β-TCP combination scaffolds were implanted into the defect areas. High-resolution micro-CT scanning was carried out to qualitatively evaluate the newly formed bone within the defects at 8 weeks post-implantation. The sagittal view of micro-CT images rarely, if at all, showed the newly regenerated bone in the pure β-TCP group (Fig. 2a). On the contrary, a large amount of de novo bone formation was observed in the calvarial defect sites following implantation with the β-TCP scaffolds loaded with 5 × 10^11^ particles/mL of exosomes. Also, a larger extent of bone regeneration was shown with an increasing exosome concentration. This was confirmed by a quantitative analysis of the BV/TV (Fig. 2b), which showed a dose-dependent increase in new bone formation in the exosome-functionalized group as compared to the control. Furthermore, the local BMDs showed the same tendency as that obtained for the BV/TV levels (Fig. 2c), indicating that hiPS-MSC-Exos could improve ossification in the defect areas.

**Fig. 3:**
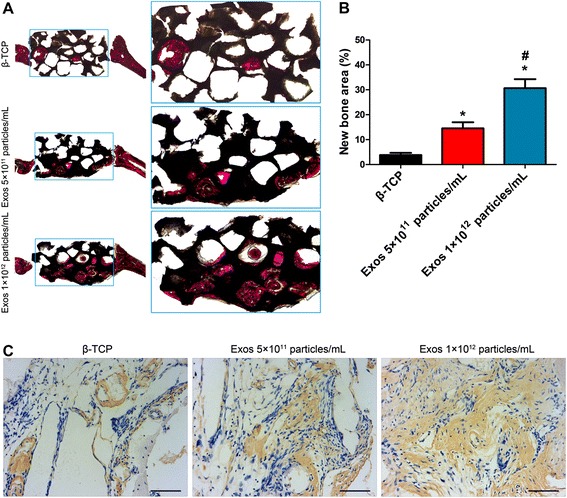
Histological and immunohistochemical analysis of the newly formed bone after 8 weeks of transplantation. **a** The un-decalcified craniums were stained with van Gieson’s picrofuchsin. The new bone area and tricalcium phosphate (*β-TCP*) residue are shown in *red* and *black*, respectively. **b** Quantitative analysis of (**a**). Dunnett t test; *p < 0.05 compared with the β-TCP group, ^#^ *p* < 0.05 compared with the exosome (*Exos*) 5 × 10^11^ particles/mL group. **c** The sections were subjected to immunohistochemical analysis to inspect the distribution of OCN. OCN-positive immunostaining illustrated the bone tissues. *Scale bars* = 80 μm

The tetracycline (yellow), alizarin red (red), and calcein (green) dyes were intraperitoneally injected into each rat at 2, 4, and 6 weeks post-operation, respectively. Results showed that the percentages of tetracycline (yellow), alizarin red (red), or calcein (green) labeling in the exosome-functionalized group were greater than those in the control group (Fig. 2d and e). Higher percentages of fluorescent dyes were observed after the bone defects were treated with a higher concentration of exosomes (Fig. 2d and e). Moreover, histological evidence based on van Gieson staining of non-decalcified craniums demonstrated that exosome treatment increased the newly formed bone tissues within the defect areas, as determined by the percentage of new bone area (Fig. 3a and b). Consistent with the above findings, OCN immunostaining further confirmed that the osteogenic responses from the defect tissues treated with exosomes was enhanced as compared to the control (Fig. 3c). Collectively, our in vivo functional studies demonstrated that hiPS-MSC-Exos could stimulate osteogenesis of β-TCP through activating a bone regenerative process in the target tissue.

### Exosome release from β-TCP

**Fig. 4:**
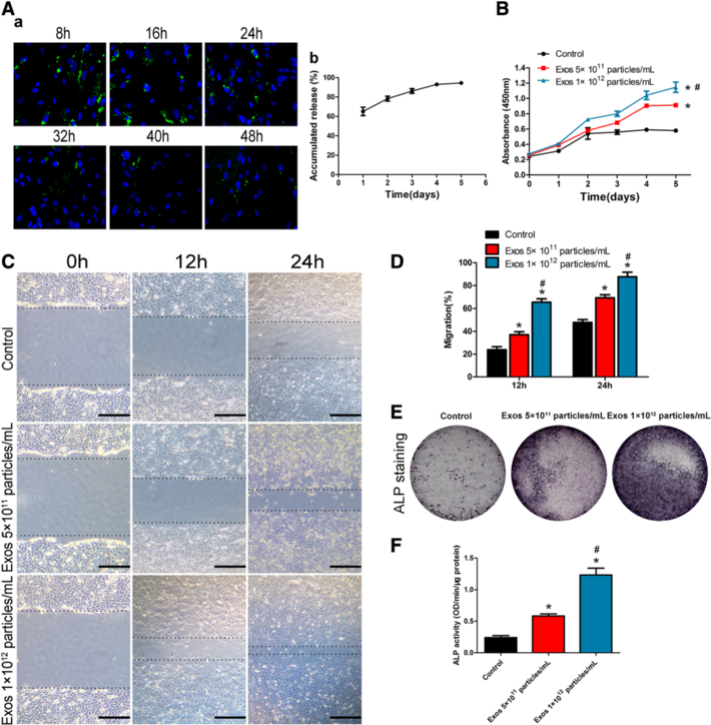
Internalization of hiPS-MSC-Exos in human BMSCs, exosome release from β-TCP, and their pro-osteogenesis effects on the recipient cells. **A** (**a**) DiO-labeled exosome release from β-TCP over 48 h. (**b**) Accumulated release of exosomes from β-TCP over 5 days. **B** Exosomes (*Exos*) enhanced the proliferation of hBMSCs as analyzed by Cell Counting Kit-8 assay. **C** Exosomes stimulated the migration of BMSCs as analyzed by migration assay. *Scale bars* = 250 μm. **D** Quantitative analysis of (**C**). **E**, **F** Incubation of hBMSCs with exosomes resulted in a dose-dependent increase in the alkaline phosphatase (*ALP*) staining and activity. Dunnett t test; *p < 0.05 compared with the control group, ^#^ *p* < 0.05 compared with the Exos 5 × 10^11^ particles/mL group

To detect exosome release from β-TCP, we used a green fluorescent lipophilic dye (DiO) to label hiPS-MSCs. The exosomes released by the labeled cells were also labeled with DiO upon fusion of multivesicular bodies with the cell plasma membrane. The labeled exosomes were combined with β-TCP and then added to the plate containing hBMSCs. The internalization of exosomes was detected every 8 h of culture with hBMSCs. The results showed that the DiO-labeled exosomes can be found in the perinuclear region of hBMSCs which surround β-TCP (Fig. 4Aa), indicating that hBMSCs are targets of hiPS-MSC-Exos.

As shown in Fig. 4Ab, the exosomes showed burst release from β-TCP. In addition, with an increase in immersed hours, exosome release decreased gradually.

### Pro-osteogenesis effects of exosomes on hBMSCs

To explore the functional roles of hiPS-MSC-Exos in osteogenesis, hBMSCs were cultured in MGro-500 MSC or osteogenic medium containing exosomes (5 × 10^11^ or 1 × 10^12^ particles/mL) or in an equal volume of PBS for a series of in vitro osteogenesis-related assays. Data indicated that 5 × 10^11^ particles/mL of exosomes were able to significantly enhance hBMSC proliferation; the hBMSCs treated with a higher concentration of exosomes (1 × 10^12^ particles/mL) showed much higher proliferative capability (Fig. 4B). As the resident MSCs are recruited into injury sites for helping repair the injury, the migration ability of MSCs is critical for bone reparation and regeneration. The scratch wound assay showed that, after being cultured with exosomes, the migration ability of hBMSCs was remarkably enhanced (Fig. 4C and D). Detection of ALP expression, a biochemical marker of osteogenesis as well as the osteocalcin content, showed that incubation of hBMSCs with hiPS-MSC-Exos resulted in a dose-dependent increase in the ALP staining (Fig. 4E) and activity (Fig. 4F), clearly indicating a positive role for hiPS-MSC-Exos in osteogenic differentiation. Taken together, our results show that hiPS-MSC-Exos could activate a series of bone regenerative responses from target hBMSCs in a dose-dependent manner. Taking economic concerns into account, and since the low dose of exosomes (5 × 10^11^ particles/mL) exert significant pro-osteogenic effects on target tissues and cells, we used the concentration of 5 × 10^11^ particles/mL for the subsequent experiments.

### Differentially expressed genes in hBMSCs stimulated by exosomes

**Fig. 5:**
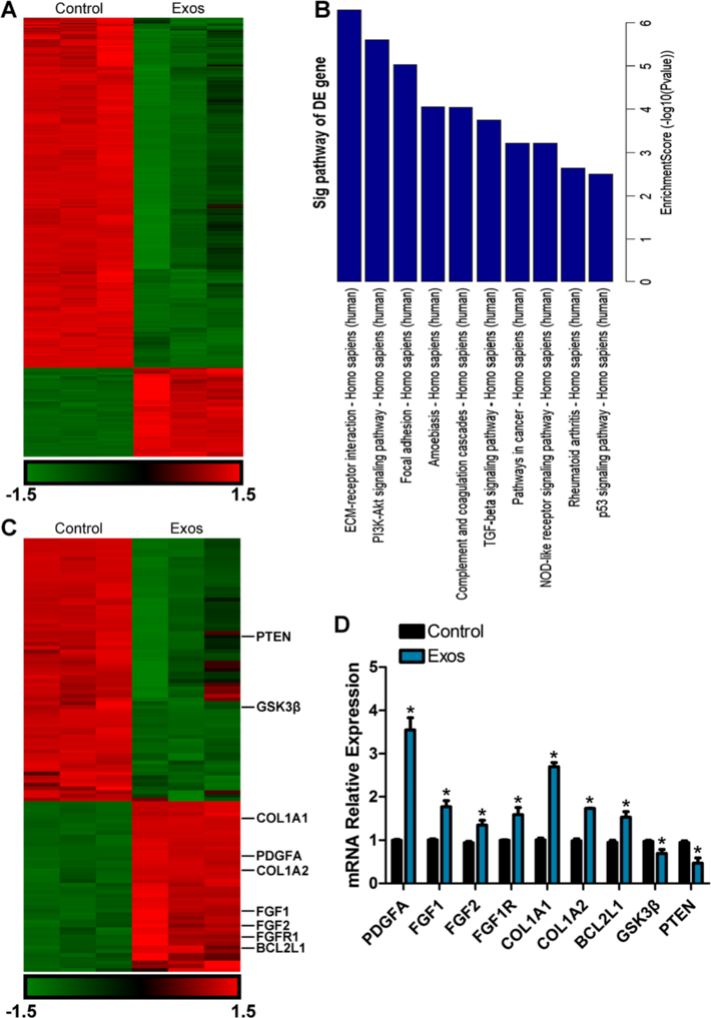
Differential expression of mRNAs between exosome-treated and control groups. **a** The differentially expressed (*DE*) genes in hBMSCs in response to exosome (*Exos*) stimulation are illustrated as a heat map. A *p* value cut-off of 0.05 and a fold-value change of ≥2 were used as a filter to identify the DE genes. **b** Enrichment analysis of all DE genes was performed. The top ten enriched pathways associated with exosome stimulation are shown. **c** A heat map of PI3K/Akt signaling pathway-related DE genes was generated. **d** The altered expression of PI3K/Akt signaling-related genes was confirmed by qRT-PCR analysis. ANOVA; *p < 0.05 compared with the control group

To determine the effector genes that respond to hiPS-MSC-Exos stimulation, microarray analyses of hBMSCs cultured with exosomes or PBS were performed. The 1.5-fold up- or downregulated genes in all three paired samples were defined as differentially expressed genes or effector genes after exposure to hiPS-MSC-Exos. The results showed that there was a significant differential expression of 1447 candidate genes between the two groups (*p* < 0.05) (Fig. 5a). Among these, 293 genes were upregulated and 1154 genes were downregulated in the exosome-treated hBMSC groups than in the control group treated with PBS. We then performed functional enrichment analysis, including molecular functions and involvement of all differentially expressed genes in known regulatory signaling pathways. The results revealed that multiple biological pathways were prominently enriched (Enrichment Score >2.0, *p* < 0.05). The top ten enriched pathways associated with exosome stimulation are shown in Fig. 5b. Among these, extracellular matrix (ECM)-receptor interaction, focal adhesion, and phosphatidylinositol 3-kinase (PI3K)-Akt signaling pathways appeared to be the most enriched ones (Enrichment Score >5.0, *p* < 0.05). Since the PI3K/Akt signaling pathway has previously been found to be involved in MSC proliferation, migration, and osteogenic differentiation [26–31], it is reasonable to believe that this signaling process might have key roles in the exosome-mediated pro-osteogenesis effects on hBMSCs.

The PI3K/Akt signaling pathway-related differentially expressed genes are illustrated as a heat map (Fig. 5c) showing that 117 genes involved in this pathway were altered in hBMSCs treated with exosomes. Among them, 47 genes, such as platelet-derived growth factor alpha (PDGFA), fibroblast growth factor 1 or 2 (FGF1/2), FGF receptor 1 (FGFR1), collagen type I alpha 1 or 2 (COL1A1/2), and BCL2-like 1 (BCL2L1), which are positive effector genes in the PI3K/Akt signaling pathway, were profoundly upregulated. Conversely, another 70 genes, such as glycogen synthase kinase 3 beta (GSK3β) and phosphatase and tensin homolog deleted on chromosome ten (PTEN) that are key molecules in negatively regulating this pathway, were markedly downregulated. These results suggested that the PI3K/Akt signaling in hBMSCs stimulated by hiPS-MSC-Exos was activated.

To confirm the microarray results, 10 genes, including VCAM1, FGF11, IL6, SLC2A1, ITGA7, LAMA1, ETNK1, TLR4, DKK1, and NKTR, which were differentially expressed between the exosome-treated BMSCs and the control group (*p* < 0.05), were randomly selected for qRT-PCR detection. The levels of VCAM1, FGF11, IL6, SLC2A1, and ITGA7 were increased in the exosome-treated hBMSCs, whereas the expression of LAMA1, ETNK1, TLR4, DKK1, and NKTR was decreased (Additional file 1: Figure S2). We also performed qRT-PCR to confirm the altered expression of PI3K/Akt signaling-associated genes. Data revealed that the exosome-treated hBMSCs showed a profound upregulation of PDGFA, FGF1/2, FGFR1, COL1A1/2, and BCL2L1, but a significant downregulation of GSK3β and PTEN (*p* < 0.05) (Fig. 5d). The results were generally consistent with the microarray data.

### Involvement of PI3K/Akt signaling in the exosome-induced osteogenic responses

**Fig. 6:**
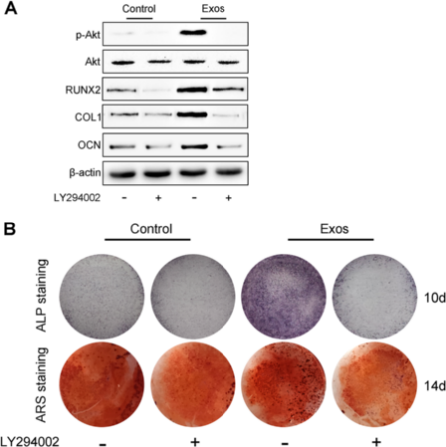
Involvement of PI3K/Akt signaling in the exosome-induced osteogenic responses from BMSCs. **a** Exosomes (*Exos*) induced the activation of the PI3K/Akt signaling pathway and increased the protein levels of osteogenesis-related molecules; these effects by exosomes were abolished by the PI3K inhibitor (LY294002; 10 μM). **b** The exosome-treated hBMSCs showed much higher levels of alkaline phosphatase (*ALP*) and Alizarin red S (*ARS*) staining compared with the control groups on days 10 and 14, respectively; these effects were inhibited by LY294002

The activation of the PI3K/Akt pathway in hBMSCs following hiPS-MSC-Exos stimulation was verified by treating the cells with exosomes or PBS for 24 h and assessing the protein levels of Akt and p-Akt by Western blotting. Compared to the PBS control, incubation with hiPS-MSC-Exos resulted in a significant increase in Akt phosphorylation on Ser473 in hBMSCs (Fig. 6a), indicating that the PI3K/Akt signaling in hBMSCs was activated by the exosomes. However, the upregulation of p-Akt in hBMSCs by the exosomes was significantly impaired after the hBMSCs were cultured with a PI3K inhibitor (LY294002; 10 μM) (Fig. 6a).

The activation of the PI3K/Akt pathway might be the underlying mechanism through which hiPS-MSC-Exos exert pro-osteogenesis effects on recipient cells. We tested this hypothesis by culturing the BMSCs in osteogenic media containing exosomes or PBS with or without LY294002. After 7 days of differentiation, Western blotting was performed to detect the levels of the early osteogenesis-related marker proteins including Runx2 and COL1, and the late osteogenic differentiation marker OCN (Fig. 6a). The results revealed that hBMSCs, when cultured in the osteogenic medium, showed osteogenic differentiation as indicated by the expression of osteogenesis-related molecules. After incubation of hBMSCs with hiPS-MSC-Exos, the levels of these osteogenesis-related proteins were further increased. However, their upregulation by hiPS-MSC-Exos was markedly suppressed by the PI3K inhibitor LY294002. Furthermore, the addition of LY294002 to the osteogenic medium alone also decreased osteogenesis slightly, suggesting that the osteogenic medium-induced osteogenesis relies on the activation of the PI3K/Akt pathway to some extent. Also, we evaluated the extent of ALP production and calcium mineral deposition in hBMSCs by staining with ALP and ARS. As shown in Fig. 6b, the exosome-treated hBMSCs showed significantly higher expression of ALP and calcium deposits than the control group, whereas these effects were blocked by the additional treatment with LY294002. The ALP generation, matrix mineralization, and calcium nodule formation were reduced slightly in BMSCs treated with LY294002 compared with the control, which was consistent with the data from osteogenesis-related molecule expression. Taken together, our results indicated that the stimulatory effects of hiPS-MSC-Exos on the osteogenic differentiation of hBMSCs mainly resulted from the activation of the PI3K/Akt signaling pathway.

## Discussion

Normal healthy bone has the ability to spontaneously regenerate after minor injury. However, extensive bone loss due to disease or trauma requires tissue engineering applications. In the present study, we provided the first demonstration that hiPS-MSC-Exos, the nanocarriers naturally secreted by iPS-MSCs, could be combined with β-TCP and this hiPS-MSC-Exos/β-TCP combination scaffold could significantly enhance osteogenesis in a rat model of critical-sized calvarial bone defects as compared to β-TCP alone. Through in vitro studies, we found that the exosomes could remarkably promote hBMSC osteogenic differentiation after they were internalized by hBMSCs through activating the PI3K/Akt signaling pathway.

Currently, a growing body of evidence indicates that MSC paracrine action may be applied as a novel strategy for tissue repair [32, 33], and exosomes are important paracrine factors that can be directly used as therapeutic agents for various disease models, such as cutaneous wound healing [24], myocardial infarction [34], traumatic brain injury [35], and immune disease [36]. However, it was unclear whether exosomes can be applied to bone tissue engineering. β-TCP is a synthetic and biodegradable ceramic material that has been commonly used for repairing bone defects, but the repair effect of pure β-TCP is very limited as it lacks osteoinductive activity. Considering the functional roles of exosomes in tissue repair, we combined exosomes with β-TCP, hoping to obtain an exosome/β-TCP composite scaffold which could enhance osteogenesis. Based on imaging and histological examinations in this study, we found that the application of exosome/β-TCP combination scaffolds in vivo could profoundly enhance bone regeneration compared to pure β-TCP. Recently, the design of surface morphological/topographies of TCP has been considered a novel approach to enhance the bioactivity and osteoinductive ability in the field of bone regeneration [6]. However, the technological process is relatively complicated and the obtained bioactivity is very limited. Bone tissue engineering has also been used, through which bone regeneration can be achieved via a combination of TCP with stem cells [4, 37]. Nevertheless, the application of stem cells is still hampered by immunological rejection, chromosomal variation, malignant transformation, and so forth [38]. The therapeutic effects of exosomes on tissue injury have been demonstrated [24, 34–36]. In addition, MSC-derived exosomes do not contain MHC class I or II proteins and, thus, their application to non-immune-compatible animals does not induce overt immune reactions [34, 39, 40]. Thus, the exosome/β-TCP combination scaffolds can be potentially used as a promising graft for bone defect repair.

It is known that endogenous BMSCs can be mobilized in response to injury, and the activated BMSCs can serve as the major healing cells during bone repair and remodeling [41]. We hypothesized that the beneficial effects of the exosome/β-TCP combination scaffolds on bone defects might be attributed to their recruitment and activation of pre-existing BMSCs in the bone tissue, and we performed in vitro experiments to confirm this hypothesis. It has been proposed that exosomes can serve as a nanocarrier to transfer proteins, mRNAs, and microRNAs from parent cells to recipient cells, and that they alter the gene expression and protein translation of the recipient cells thereby modulating the bioactivity of target cells [42]. Their internalization by target cells is the prerequisite for playing such a role. In the present study, the DiO-labeled exosomes were combined with β-TCP, and then put into cell culture plates containing hBMSCs. The labeled exosomes can be seen in perinuclear regions of hBMSCs, indicating exosomes could release from exosome/β-TCP combination scaffolds and then be internalized by hBMSCs. Further analysis showed that, after internalization of exosomes into hBMSCs, they stimulate the proliferation, migration, and osteogenic differentiation of hBMSCs. These conditions are beneficial for attracting endogenous BMSCs to bone defects and finally enhancing new bone formation. All findings demonstrated that the exosome/β-TCP combination scaffolds have obviously enhanced osteogenic activity attributed to the biological effects of exosomes.

We further clarified the molecular mechanisms of exosome/β-TCP combination scaffolds on the osteogenesis of hBMSCs. Zhang et al. reported that the exosomes released by human umbilical cord blood-derived MSCs were able to induce β-catenin nuclear translocation of skin cells, enhancing their proliferation and migration [43]. Shabbir et al. showed that the exosomes secreted by human BMSCs could activate several signaling pathways (including Akt, Erk1/2, and STAT3) in target fibroblasts and increase their angiogenic response [44]. In this study, we used microarray analysis to examine the gene expression pattern in hBMSCs treated by hiPS-MSC-Exos. We detected significant alterations in the expression of a variety of genes following treatment with exosomes. Results of bioinformatics analyses indicated that these significantly altered genes are predominantly involved in the PI3K/Akt signaling pathway, focal adhesion, and ECM-receptor interaction. In particular, the PI3K/Akt pathway may have a role in the exosome-induced pro-osteogenic effects on hBMSCs as this signaling cascade has been reported to play important roles in osteoblast differentiation and bone growth [26–28]. Here, we report that hiPS-MSC-Exos treatment resulted in a prominent increase in the expression of the positive effector genes of the PI3K/Akt pathway, such as PDGFA, FGF1/2, FGFR1, COL1A1/2, and BCL2L1. All of these genes can positively modulate cell survival, proliferation, migration, osteogenic differentiation, or apoptotic inhibition [29–31, 45, 46]. The typical negative modulators of PI3K/Akt signaling, including GSK3β and PTEN, were remarkably decreased. The results indicated that the PI3K/Akt signaling in hBMSCs is probably activated after internalization of exosomes. This contention was confirmed by a significant increase in the phosphorylation of Akt (an indicator of PI3K activation) in hBMSCs treated with exosomes. However, it is noteworthy that the PI3K inhibitor markedly, but not completely, abolished the pro-osteogenic effects of hiPS-MSC-Exos, suggesting that the exosome-induced osteogenic effects cannot be fully attributed to the PI3K/Akt pathway and therefore additional mechanisms remain to be explored. Our future studies will focus on deciphering the molecular contents of hiPS-MSC-Exos that underlie the activation of the PI3K/Akt signaling pathway that appears to be largely responsible for the osteogenesis in hBMSCs.

## Conclusions

In this study, we provided evidence that the hiPS-MSCs-Exos-functionalized β-TCP scaffold can effectively promote bone repair and regeneration in a rat model of calvarial bone defects. The underlying mechanism through which exosomes enhance osteoinductive activity of β-TCP and promote bone regeneration appears to be the activation of endogenous BMSCs in the bone defect site. This was suggested by the observation that exosomes could release from exosome/β-TCP combination scaffolds and then be internalization by hBMSCs. Furthermore, the PI3K/Akt signaling pathway likely plays a critical role in the pro-osteogenesis effects of the exosome/β-TCP scaffold on BMSCs. Further studies are needed to assess the long-term effects of exosome-based therapy for bone repair and whether the exosome-functionalized β-TCP can be used as a valid and safe therapeutic option for clinical application.

## Additional file

Additional file 1: Table S1. Primers used for quantitative real-time PCR (qRT-PCR) analysis. **Figure S1.** Characterization of hiPS-MSCs. (A) Flow cytometry analysis of the cell surface markers in hiPS-MSCs. (B) The qRT-PCR results for OCN, Sox9, and LPL after 7 days in culture with osteo-, chondro-, and adipogenic mediun. ANOVA, **p* < 0.05 compared with the control group. **Figure S2.** Validation of mRNA profiles using qRT-PCR on the same sample set of microarray study. Ten differentially expressed genes were randomly selected from the microarray data sets for qRT-PCR analysis. (DOCX 280 kb)

## Abbreviations

ALP: Alkaline phosphatase
ARS: Alizarin red S
BMD: Bone mineral density
CCK-8: Cell Counting Kit-8
hiPS-MSCs: Human induced pluripotent stem cell-derived mesenchymal stem cells
IHC: Immunohistochemical
OCN: Osteocalcin
OD: Optical density
PMMA: Polymethylmethacrylate
TEM: Transmission electron microscopy
TRPS: Tunable resistive pulse sensing

## References

[1] Arrington ED, Smith WJ, Chambers HG, Bucknell AL, Davino NA. Complications of iliac crest bone graft harvesting. Clin Orthop Relat Res. 1996;(329):300–9. 10.1097/00003086-199608000-00037 8769465

[2] Berrey BH, Lord CF, Gebhardt MC, Mankin HJ (1990). Fractures of allografts. Frequency, treatment, and end-results. J Bone Joint Surg Am.

[3] Fujibayashi S, Kim HM, Neo M, Uchida M, Kokubo T, Nakamura T (2003). Repair of segmental long bone defect in rabbit femur using bioactive titanium cylindrical mesh cage. Biomaterials.

[4] Zhou H, Xiao C, Wang Y, Bi X, Ge S, Fan X (2011). In vivo efficacy of bone marrow stromal cells coated with beta-tricalcium phosphate for the reconstruction of orbital defects in canines. Invest Ophthalmol Vis Sci.

[5] Katagiri W, Osugi M, Kawai T, Hibi H (2016). First-in-human study and clinical case reports of the alveolar bone regeneration with the secretome from human mesenchymal stem cells. Head Face Med.

[6] Yuan H, Fernandes H, Habibovic P, de Boer J, Barradas AM, de Ruiter A (2010). Osteoinductive ceramics as a synthetic alternative to autologous bone grafting. Proc Natl Acad Sci U S A.

[7] Zhang J, Guan J, Zhang C, Wang H, Huang W, Guo S (2015). Bioactive borate glass promotes the repair of radius segmental bone defects by enhancing the osteogenic differentiation of BMSCs. Biomed Mater.

[8] Han P, Xu M, Chang J, Chakravorty N, Wu C, Xiao Y (2014). Lithium release from beta-tricalcium phosphate inducing cementogenic and osteogenic differentiation of both hPDLCs and hBMSCs. Biomater Sci..

[9] Guan J, Zhang J, Zhu Z, Niu X, Guo S, Wang Y (2015). Bone morphogenetic protein 2 gene transduction enhances the osteogenic potential of human urine-derived stem cells. Stem Cell Res Ther..

[10] Kamolz LP, Keck M, Kasper C (2014). Wharton’s jelly mesenchymal stem cells promote wound healing and tissue regeneration. Stem Cell Res Ther.

[11] Liang X, Ding Y, Zhang Y, Tse HF, Lian Q (2014). Paracrine mechanisms of mesenchymal stem cell-based therapy: current status and perspectives. Cell Transplant.

[12] Baglio SR, Pegtel DM, Baldini N (2012). Mesenchymal stem cell secreted vesicles provide novel opportunities in (stem) cell-free therapy. Front Physiol..

[13] Hu GW, Li Q, Niu X, Hu B, Liu J, Zhou SM (2015). Exosomes secreted by human-induced pluripotent stem cell-derived mesenchymal stem cells attenuate limb ischemia by promoting angiogenesis in mice. Stem Cell Res Ther..

[14] De Jong OG, Van Balkom BW, Schiffelers RM, Bouten CV, Verhaar MC (2014). Extracellular vesicles: potential roles in regenerative medicine. Front Immunol..

[15] Nakamura Y, Miyaki S, Ishitobi H, Matsuyama S, Nakasa T, Kamei N (2015). Mesenchymal-stem-cell-derived exosomes accelerate skeletal muscle regeneration. FEBS Lett.

[16] Zhang B, Yin Y, Lai RC, Tan SS, Choo AB, Lim SK (2014). Mesenchymal stem cells secrete immunologically active exosomes. Stem Cells Dev.

[17] Jiang ZZ, Liu YM, Niu X, Yin JY, Hu B, Guo SC (2016). Exosomes secreted by human urine-derived stem cells could prevent kidney complications from type I diabetes in rats. Stem Cell Res Ther.

[18] Burger D, Vinas JL, Akbari S, Dehak H, Knoll W, Gutsol A (2015). Human endothelial colony-forming cells protect against acute kidney injury: role of exosomes. Am J Pathol.

[19] Xin H, Li Y, Chopp M (2014). Exosomes/miRNAs as mediating cell-based therapy of stroke. Front Cell Neurosci..

[20] Doeppner TR, Herz J, Gorgens A, Schlechter J, Ludwig AK, Radtke S (2015). Extracellular vesicles improve post-stroke neuroregeneration and prevent postischemic immunosuppression. Stem Cells Transl Med.

[21] Lian Q, Zhang Y, Zhang J, Zhang HK, Wu X, Zhang Y (2010). Functional mesenchymal stem cells derived from human induced pluripotent stem cells attenuate limb ischemia in mice. Circulation.

[22] Fu QL, Chow YY, Sun SJ, Zeng QX, Li HB, Shi JB (2012). Mesenchymal stem cells derived from human induced pluripotent stem cells modulate T-cell phenotypes in allergic rhinitis. Allergy.

[23] Villa-Diaz LG, Brown SE, Liu Y, Ross AM, Lahann J, Parent JM (2012). Derivation of mesenchymal stem cells from human induced pluripotent stem cells cultured on synthetic substrates. Stem Cells.

[24] Zhang J, Guan J, Niu X, Hu G, Guo S, Li Q (2015). Exosomes released from human induced pluripotent stem cells-derived MSCs facilitate cutaneous wound healing by promoting collagen synthesis and angiogenesis. J Transl Med..

[25] Zou L, Luo Y, Chen M, Wang G, Ding M, Petersen CC (2013). A simple method for deriving functional MSCs and applied for osteogenesis in 3D scaffolds. Sci Rep..

[26] Ghosh-Choudhury N, Abboud SL, Nishimura R, Celeste A, Mahimainathan L, Choudhury GG (2002). Requirement of BMP-2-induced phosphatidylinositol 3-kinase and Akt serine/threonine kinase in osteoblast differentiation and Smad-dependent BMP-2 gene transcription. J Biol Chem.

[27] Fujita T, Azuma Y, Fukuyama R, Hattori Y, Yoshida C, Koida M (2004). Runx2 induces osteoblast and chondrocyte differentiation and enhances their migration by coupling with PI3K-Akt signaling. J Cell Biol.

[28] Suzuki E, Ochiai-Shino H, Aoki H, Onodera S, Saito A, Saito A (2014). Akt activation is required for TGF-beta1-induced osteoblast differentiation of MC3T3-E1 pre-osteoblasts. PLoS One.

[29] Yokota J, Chosa N, Sawada S, Okubo N, Takahashi N, Hasegawa T (2014). PDGF-induced PI3K-mediated signaling enhances the TGF-beta-induced osteogenic differentiation of human mesenchymal stem cells in a TGF-beta-activated MEK-dependent manner. Int J Mol Med.

[30] Radcliff K, Tang TB, Lim J, Zhang Z, Abedin M, Demer LL (2005). Insulin-like growth factor-I regulates proliferation and osteoblastic differentiation of calcifying vascular cells via extracellular signal-regulated protein kinase and phosphatidylinositol 3-kinase pathways. Circ Res.

[31] Debiais F, Lefevre G, Lemonnier J, Le Mee S, Lasmoles F, Mascarelli F (2004). Fibroblast growth factor-2 induces osteoblast survival through a phosphatidylinositol 3-kinase-dependent, -beta-catenin-independent signaling pathway. Exp Cell Res.

[32] Osugi M, Katagiri W, Yoshimi R, Inukai T, Hibi H, Ueda M (2012). Conditioned media from mesenchymal stem cells enhanced bone regeneration in rat calvarial bone defects. Tissue Eng Part A.

[33] Wang KX, Xu LL, Rui YF, Huang S, Lin SE, Xiong JH (2015). The effects of secretion factors from umbilical cord derived mesenchymal stem cells on osteogenic differentiation of mesenchymal stem cells. PLoS One.

[34] Lai RC, Arslan F, Lee MM, Sze NS, Choo A, Chen TS (2010). Exosome secreted by MSC reduces myocardial ischemia/reperfusion injury. Stem Cell Res.

[35] Zhang Y, Chopp M, Meng Y, Katakowski M, Xin H, Mahmood A (2015). Effect of exosomes derived from multipluripotent mesenchymal stromal cells on functional recovery and neurovascular plasticity in rats after traumatic brain injury. J Neurosurg.

[36] Rahman MJ, Regn D, Bashratyan R, Dai YD (2014). Exosomes released by islet-derived mesenchymal stem cells trigger autoimmune responses in NOD mice. Diabetes.

[37] Zou D, Zhang Z, He J, Zhu S, Wang S, Zhang W (2011). Repairing critical-sized calvarial defects with BMSCs modified by a constitutively active form of hypoxia-inducible factor-1alpha and a phosphate cement scaffold. Biomaterials.

[38] Wang Y, Han ZB, Song YP, Han ZC (2012). Safety of mesenchymal stem cells for clinical application. Stem Cells Int..

[39] Lai RC, Arslan F, Tan SS, Tan B, Choo A, Lee MM (2010). Derivation and characterization of human fetal MSCs: an alternative cell source for large-scale production of cardioprotective microparticles. J Mol Cell Cardiol.

[40] Lai RC, Yeo RW, Tan KH, Lim SK (2013). Mesenchymal stem cell exosome ameliorates reperfusion injury through proteomic complementation. Regen Med.

[41] Deschaseaux F, Sensebe L, Heymann D (2009). Mechanisms of bone repair and regeneration. Trends Mol Med.

[42] Camussi G, Deregibus MC, Bruno S, Cantaluppi V, Biancone L (2010). Exosomes/microvesicles as a mechanism of cell-to-cell communication. Kidney Int.

[43] Zhang B, Wang M, Gong A, Zhang X, Wu X, Zhu Y (2015). HucMSC-exosome mediated-Wnt4 signaling is required for cutaneous wound healing. Stem Cells.

[44] Shabbir A, Cox A, Rodriguez-Menocal L, Salgado M, Van Badiavas E (2015). Mesenchymal stem cell exosomes induce proliferation and migration of normal and chronic wound fibroblasts, and enhance angiogenesis in vitro. Stem Cells Dev.

[45] Tsai KS, Kao SY, Wang CY, Wang YJ, Wang JP, Hung SC (2010). Type I collagen promotes proliferation and osteogenesis of human mesenchymal stem cells via activation of ERK and Akt pathways. J Biomed Mater Res A.

[46] Zhou F, Yang Y, Xing D (2011). Bcl-2 and Bcl-xL play important roles in the crosstalk between autophagy and apoptosis. FEBS J.

