# Supplementary material for: Exosomes/tricalcium phosphate combination scaffolds can enhance bone regeneration by activating the PI3K/Akt signaling pathway
Source: Stem Cell Res Ther. 2016 Sep 20;7:136. doi: 10.1186/s13287-016-0391-3 (PMC5028974; doi:10.1186/s13287-016-0391-3)
Supplement: Additional file 1: Table S1. — Primers used for quantitative real-time PCR (qRT-PCR) analysis. Figure S1. Characterization of hiPS-MSCs. (A) Flow cytometry analysis of the cell surface markers in hiPS-MSCs. (B) The qRT-PCR results for OCN, Sox9, and LPL after 7 days in culture with osteo-, chondro-, and adipogenic mediun. ANOVA, *p < 0.05 compared with the control group. Figure S2. Validation of mRNA profiles using qRT-PCR on the same sample set of microarray study. Ten differentially expressed genes were randomly selected from the microarray data sets for qRT-PCR analysis. (DOCX 280 kb) [file 13287_2016_391_MOESM1_ESM.docx]

**Supporting information for**

**Exosomes/tricalcium phosphate combination scaffolds can enhance bone regeneration by activating PI3K/Akt signaling pathway**

Jieyuan Zhang^1,2†^, Xiaolin Liu^1,2†^, Haiyan Li^3^, Chunyuan Chen^4^, Bin Hu^1^, Xin Niu^1^, Qing Li^1^, Bizeng Zhao^2^, Zongping Xie^2*^, Yang Wang^1*^

^1^Institute of Microsurgery on Extremities, Shanghai Jiao Tong University Affiliated Sixth People's Hospital, 600 Yishan Road, Shanghai 200233, China.

^2^Department of Orthopedic Surgery, Shanghai Jiao Tong University Affiliated Sixth People's Hospital, 600 Yishan Road, Shanghai 200233, China.

^3^Med-X Research Institute, School of Biomedical Engineering, Shanghai Jiao Tong University, 1954 Huashan Road, Shanghai 200030, China.

^4^Graduate School of Nanchang University, 461 Bayi Road, Nanchang 330006, China.

*Correspondence: wangy63cn@126.com; x91034@qq.com

†Equal contributors

**Table S1**

**Table S1** **Primers used for quantitative real-time PCR (qRT-PCR) analysis**

| Genes | Forward Primer (5’-3’) | Reverse Primer (5’-3’) |
| --- | --- | --- |
| *PDGFA* | TCCGTAGGGAGTGAGGATTCTT | AATGACCGTCCTGGTCTTGC |
| *FGF1* | CAGTGGATGGGACAAGGGAC | GGTGTTGAGCCGTATAAAAGCC |
| *FGF2* | CAATTCCCATGTGCTGTGAC | ACCTTGACCTCTCAGCCTCA |
| *FGFR1* | GGCAGTGACACCACCTACTT | GCTACGGGCATACGGTTTG |
| *COL1A1* | CACTGGTGATGCTGGTCCTG | CGAGGTCACGGTCACGAAC |
| *COL1A2* | CAGCCGGAGATAGAGGACCA | CAGCAAAGTTCCCACCGAGA |
| *BCL2L1* | TTCAGTGACCTGACATCCCA | CTGCTGCATTGTTCCCATAG |
| *GSK3β* | GACTAAGGTCTTCCGACCCC | TTAGCATCTGACGCTGCTGT |
| *PTEN* | CAAGATGATGTTTGAAACTATTCCAATG | CCTTTAGCTGGCAGACCACAA |
| *VCAM1* | TGGGAAAAACAGAAAAGAGGTG | GTCTCCAATCTGAGCAGCAA |
| *FGF11* | GCGGCACCAAGTCCCTTT | TTTGAGCTGAGGCTCCGG |
| *IL6* | AGTTCCTGCAGAAAAAGGCAAAG | AAAGCTGCGCAGAATGAGAT |
| *SLC2A1* | TCACTGTCGTGTCGCTGTTT | ACGATGAACCATGGGATGGG |
| *ITGA7* | CCTCTTCGGCTTCTCTGTGG | GCATATCAGCTCCCTGGTCG |
| *LAMA1* | AATGGTGCTGGCAGGATAAC | CCAGGACAGGAATGAAGGAA |
| *ETNK1* | CTGCGTGCTTACCTTGAAGC | ACGAACAATTGCATACCCAAGG |
| *TLR4* | CGATTCCATTGCTTCTTG | GCTCAGGTCCAGGTTCTT |
| *DKK1* | ATTCCAACGCTATCAAGAACC | CCAAGGTGCTATGATCATTACC |
| *NKTR* | ACAGTCGATCCAGGTCCTAC | GGCTCCGGTGATGAGAGTAA |
| *GAPDH* | ATCCCATCACCATCTTCC | GAGTCCTTCCACGATACCA |

**Fig. S1**

**
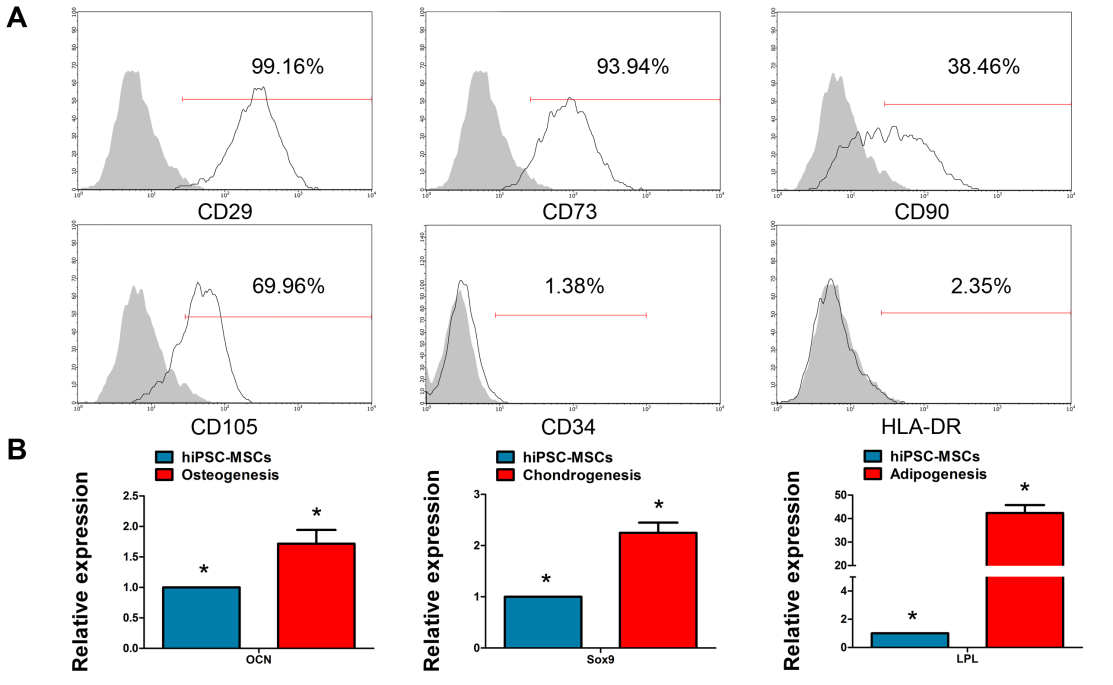
**

**Fig. S1** Characterization of hiPS-MSCs: (A) Flow cytometry analysis of the cell surface markers in hiPS-MSCs. (B) The qRT-PCR results for OCN, Sox9, and LPL after 7 days in culture with osteo-, chondro-, and adipogenic mediun. (ANOVA, **p* < 0.05 compared with the control group)

**Fig. S2**

**
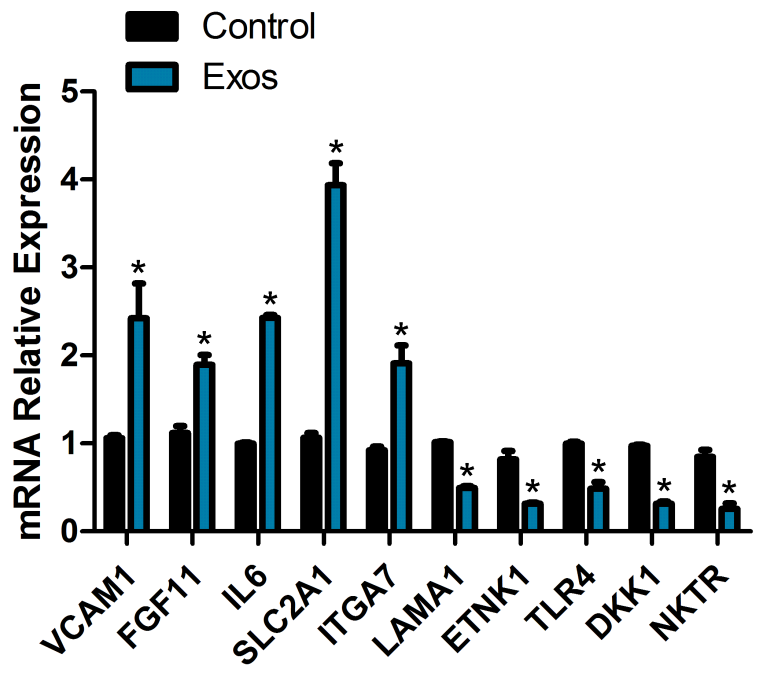
**

**Fig. S2** Validation of mRNA profiles using qRT-PCR on the same sample set of microarray study. Ten differentially expressed genes were randomly selected from the microarray data sets for qRT-PCR analysis. (ANOVA, **p* < 0.05 compared with the control group)
